# Dynamics of HeHHe^+^ Rotational State Changes Induced by Collision with He: A Possible New Path in Early Universe Chemistry

**DOI:** 10.1021/acs.jpca.1c01820

**Published:** 2021-04-26

**Authors:** L. González-Sánchez, E. Yurtsever, R. Wester, F. A. Gianturco

**Affiliations:** † Departamento de Química Física, University of Salamanca, Plaza de los Caídos sn, 37008 Salamanca, Spain; ‡ Department of Chemistry, Koc University, Rumelifeneri Yolu, Sariyer, 34450 Istanbul, Turkey; § Institut fur Ionen Physik und Angewandte Physik, Leopold-Franzens-Universitat, Technikerstrasse 25, 6020 Innsbruck, Austria

## Abstract

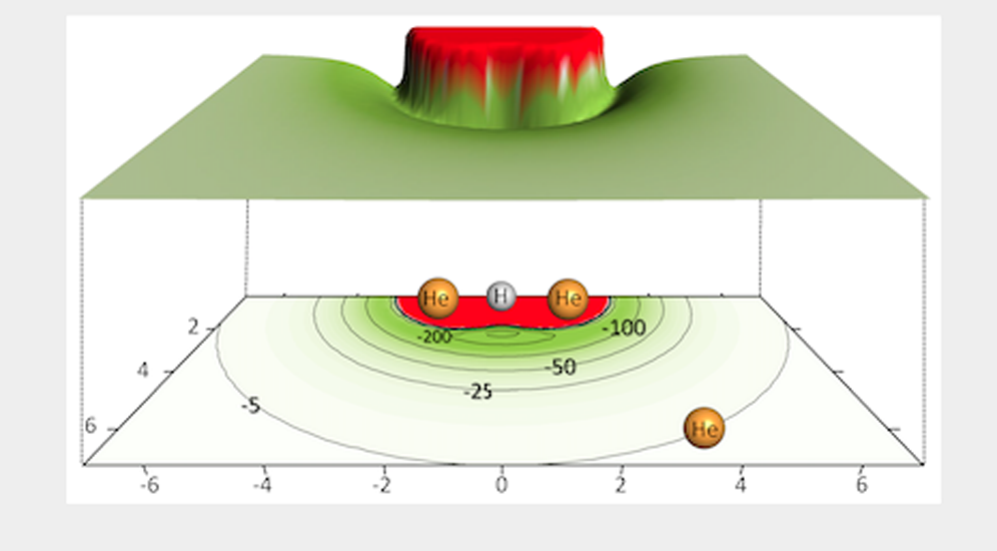

Ab initio calculations are employed to generate the rigid rotor (RR) potential energy surface (PES) describing the interaction of the linear molecular cation HeHHe^+^, at its equilibrium geometry, with the neutral He atom. The resulting interaction is employed to investigate the efficiency of rotational state-changing collisions at the temperatures relevant to the early universe conditions, where the latter molecule has been postulated to exist, albeit not yet observed. The inelastic rate coefficients are found to be fairly large and are compared with those found for another important cation just recently observed in the interstellar medium: the HeH^+^ polar molecule. The possibility for this cation to provide new options to energy dissipation routes under early universe conditions after the recombination era is briefly discussed.

## Introduction

Chemical species made up of He and H atoms are known to be strongly bound when existing as cations so that the possible series of polyatomic structures of the HeH_*n*_^+^ are all fairly stably bound and also happen to be composed of the two most abundant elements of our universe.^1,2^ In fact, the strongly bound HHe^+^ cation, which is thought to be the very first diatomic molecule formed when the early universe was cooling down,^3,4^ has been known to exist in laboratories since 1925^5^ and has been investigated by high-resolution vibrational^6−9^ and rotational^10,11^ spectroscopy studies quite extensively. Such detailed studies, in fact, have finally enabled the detection of HHe^+^ in interstellar space, an achievement that has been recently reached thanks to the GREAT receiver on-board the SOFIA airplane-borne detector.^12,13^

Several results from mass spectrometric studies^14,15^ and from high-level first-principles quantum-chemical computations^1,16−18^ indicate that the larger HHe_*n*_^+^ species (with *n* = 2–6) are known to be bound and to consist of a relatively strongly bound HeH^+^He core, with a dissociation energy of *D*_0_ = 3931 ± 20 cm^–1^ (refs (2, 16)), while additional He atoms are more loosely bound to the central proton by only about 200 cm^–1^.

The analysis of the properties of the first two terms of the series, HeH^+^ and HeHHe^+^, is a relevant issue within the context of their possible presence and activity within the interstellar medium (ISM) or within the chemistry of the early universe conditions. The knowledge of their structural properties, as well as their spectroscopic properties,^19^ has therefore been pursued extensively over the years, as discussed before in the literature and will be further illustrated below. Under the expected conditions of the early universe, which are mostly provided by the results from modeling studies (e.g., see refs (3, 13)), the chemical networks have to rely on the first two elements of the periodic table as the richness of chemistry would only come later when early stars would turn ^1^H and ^4^He (along with ^2^H and ^3^He) into carbon, nitrogen, oxygen, and the rest of the broad panoply of species observed today.^20,21^ It is also interesting to note that molecular gases subjected to intense X-rays were found to be profoundly modified by the extensive ionization of its atomic components^22^ so that it stands to reason to expect the presence of highly reactive species like He^+^ and He_2_^+^ ions and that they in turn could drive primordial chemistry with H and H^+^ or H_2_ and H_2_^+^.

The structural and spectroscopic properties of HeHHe^+^ have been recently examined and discussed in the current literature by various authors,^23,24^ where specific experimental identification has been carried out for that same molecule.^25^ We therefore have now a fair amount of information on its likely structure and possible spectroscopic signatures. Furthermore, the formation of HeHHe^+^ has been suggested to take place through reactions of He_2_^+^ with H_2_, with the ejection of a hydrogen atom^26^ and the formation of the linear, centro-symmetric HeHHe^+^ molecule in addition to other cations such as HeH^+^ and HeH_2_^+^.^27^ Detailed, possible pathways to the creation and destruction in the ISM of this linear cation have been recently analyzed in ref (23), where different interesting possibilities were put forward as to the formation of this cation.

In the analysis of the primordial chemistry, largely based on numerical models, which are still under refinement or confirmation (e.g., see ref (21)), it was suggested that, when the reacting species with neutral H would be provided as He_2_^+^ or He^+^, the latter ions would be present mainly at high redshifts and at high temperatures since the ensuing expansion and cooling of the primordial gas would efficiently lead to their associative neutralization. On the other hand, a formation channel involving HeH^+^ with He would be a possible process favored at lower *T* values, where neutral He atoms would be present. However, there is an epoch of a few thousand years where He is present and neutral H is not. It would therefore be interesting to know if the HeH^+^ + He → HeHHe^+^ reaction could actually form, in quantity, enough amount of the proton-bound complex before the competing reaction: HeH^+^ + H → H_2_^+^ + He starts taking over and hence favor the destruction of one of the important partners for the HeHHe^+^-forming chemistry.

Either way, the possible positive outcome which would be of interest for the present study would be that the formation of a very stable, strongly bound linear cation would then have a reasonable chance to survive long enough to contribute to the general dynamical network of molecular energy redistribution and dissipation during the cooling stages of the early universe environment after the recombination era.^28^

In the present work, we therefore intend to investigate as accurately as possible the dynamical option of significant changes of the internal rotational energy content of the title molecule when it interacts with the neutral He atoms present in the same environment via eq 1
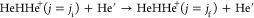
1

where the process could lead to either rotational excitation (internal heating) of the cation or its deexcitation (internal cooling) with energy dissipation into the environment. More specifically, we intend to start obtaining from first principles the interaction potential between the ionic molecular partner and the neutral He atom. The latter potential energy surface (PES) will then be employed to calculate the quantum inelastic cross sections and rate coefficients for the rotational states of the present cation on collision with neutral helium. A comparison will also be shown with the existing data on the behavior of the HeH^+^ cation, the one which has been already detected in the ISM ambient, in collision with both He and H atoms. The present new data could thus be employed for upgrading existing kinetic networks with the inclusion of the state-changing rate constants for this new molecular partner within the early universe chemical scenario.

## Methods

### Ab Initio Calculations for the He/HeHHe^+^ PES

Calculations were carried out using a variety of post-Hartree–Fock ab initio methods. In one level of analysis, all of the molecular species involved are fully optimized using the coupled-cluster method with full treatment of singles and doubles and an iterative treatment of triples: CCSD(T) as implemented in the MOLPRO suite of codes.^29,30^ The basis set is chosen to be aug-cc-pVTZ, which was further extended to aVQZ and aV5Z to achieve the complete basis set (CBS) expansion. The harmonic zero-point-energy (ZPE) corrections were included in all of these calculations.

The structural parameters of the molecular partner produced by our calculations yielded a He–H bond distance of 0.9249 Å and therefore an overall He–He distance of 1.8498 Å. These values are in good accordance with those reported in ref (23). The two-dimensional (2D) potential energy surface was sampled by mapping the (*R*,θ) space over 10 evenly spaced angles from 0 to 90° and over radial distances from 2.05 to 20.00 Å, for a total of 750 points. The angle θ is between the molecular bond and the radial distance from the center of mass of the cation (i.e., the H atom) and the impinging He atom. The full PES was then obtained by duplication of the points from 90 to 180°, thereby mapping it with a total of about 1500 points. When the angular sectors for additional angles, beyond the initial set mentioned earlier, were added to test the calculations, we found that the ensuing scattering expansion (see below) did not change significantly.

**Figure 1:**
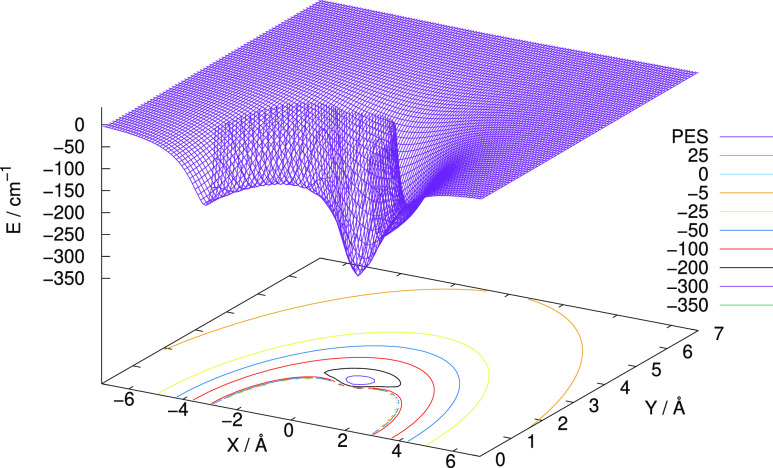
3D presentation of the PES for the title molecular cation and the neutral He atom. The origin of the coordinates is taken at the center of mass of the molecular ion.

We report in Figure 1 a 3D representation of our computed interaction, which provides a pictorial and clear view of the marked anisotropy of the present PES, a feature of the interaction which will be important when discussing the quantum dynamics of the rotational energy transfer collisions. We also see that the most stable structure of the complex corresponds to the *T*-shaped geometry with the incoming neutral He pointing at the H atom at the center of mass of the partner cation, as expected from simple electrostatics, given the location of the positive charge in the cation. The present value found for that well depth is about −350.0 cm^–1^ at a distance of about 1.137 Å.

To gain additional information on the scattering-oriented features of the present PES, we have also expanded the potential in terms of Legendre polynomials
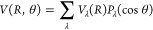
2

**Figure 2:**
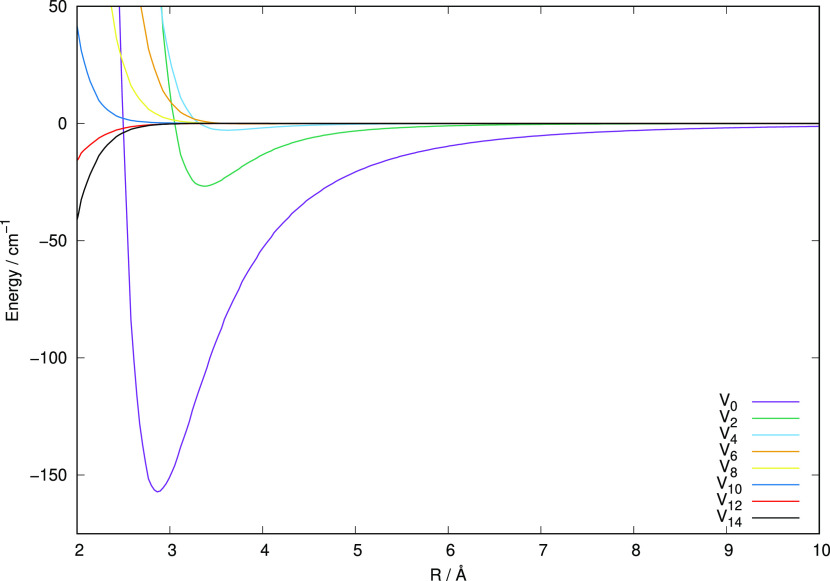
Multipolar expansion coefficients for the RR-PES potential computed in the present study.

The resulting expansion coefficients (*V*_λ_’s) provide radial functions, which are directly linked, in the following section, to the angular dynamical torque generated by the present PES during the collisions, the one causing changes of the initial rotational states of the cation. Some of these radial coefficients are plotted in Figure 2, which shows the relative strength of each of them over the expected radial range of action of the potential. The dominant attractive features shown by the λ = 0 isotropic coefficient reflect one important aspect of the physics of the interaction of the cation, which involves the spherical polarizability of the He partner, leading to the possible formation of bound complexes. We shall not be discussing here the mechanism of formation of such complexes as they will be the subject of a different publication dealing with the formation mechanisms of the title cation.

On the other hand, the significant attractive wells shown by the radial λ = 2 and 4 anisotropic coefficients indicate that rotational transitions involving Δ*j* = 2 and 4 will be more efficiently activated during collisions since their angular coefficients would directly couple the *j* and *j* ± 2,4 rotational levels. Hence, the data in Figure 2 allow us to explicitly judge the relative strength of the multipolar coefficients of the 2D PES in dynamically directing flux into the different inelastic final channels. The actual numerical raw grid of the individual radial coefficients has been interpolated to obtain a polynomial representation that was further expanded asymptotically, within the employed scattering code, to correctly represent the spherical and dipolar leading terms of the long-range interaction. An additional exponential extrapolation was employed to reach shorter distances in the repulsive region.

**Figure 3:**
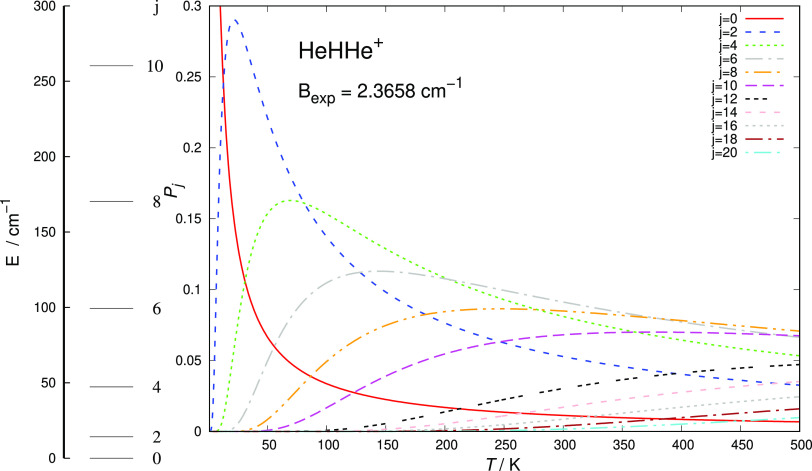
Boltzmann-averaged distributions of the rotational states of the HeHHe^+^ cation as a function of temperature. The rotational constant employed for the averaging is reported.

It is also of interest to look at the properties of the HeHHe^+^ molecular ion in terms of the energy spacings between its lower rotational levels and the Boltzmann-averaged distributions of their relative populations over the significant range of temperatures for the present processes. The results are reported in Figure 3, where one sees how many of the rotational states can be populated under equilibrium conditions up to 500 K. Note that the symmetry of the present rotor only allows for even values of the rotational quantum number *j*. This range of *T* values will be the actual range of interest for the low redshift regimes, where neutral He is expected to be more present.^21^ These features will also play a role when discussing, in the following section, the results from the quantum dynamics and the relative behavior of the different state-changing collisional rate constants between the rotational states of the title cation.

The following section shall report a short outline of the quantum method we have employed to calculate inelastic cross sections and rate coefficients for the present system.

### Quantum Dynamics for Rotational State-Changing Collisions

The standard time-independent formulation of the coupled-channel (CC) approach to quantum molecular scattering will not be repeated here in detail (see, for example, ref (31) for a general formulation) since the general literature on the actual computational methods has been very extensive. For a selected set of references over the more recent years, see, for instance, refs (32−36). Since we have already discussed the method we employ in many of our earlier publications,^37−39^ only a short outline will be given below.

For processes where no important chemical modifications would occur in the molecule by the interaction with this impinging projectile at the energies of interest, the total scattering wave function can be expanded in terms of asymptotic target rotational eigenfunctions (within the rigid rotor approximation), which are taken to be spherical harmonics and whose eigenvalues are given by *Bj*(*j* + 1), where *B* is the rotational constant for the closed-shell HeHHe^+^ anion: 2.3658 cm^–1^ as given in ref (23). The channel components for the CC equations are therefore expanded into products of total angular momentum *J* eigenfunctions and of radial functions to be determined via the solutions of the CC equations,^37,38^ i.e., the familiar set of coupled, second-order homogeneous differential equations
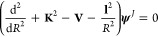
3

In the above equation, the matrices **K**^2^, **V**, and **l**^2^, respectively, represent the kinetic energy channel values, the coupling potential terms, and the centrifugal angular momenta values for each of the coupled channels in the equations.

A variety of scattering observables are therefore obtained in the asymptotic region of the interaction between partners, where the log-derivative matrix has a known form in terms of free-particle solutions and unknown mixing coefficients.^31^ Therefore, at the end of the propagation of the radial solutions from the coupled equations, one can use the log-derivative matrix to obtain the K-matrix by solving the following linear system
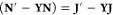
4where **J**(*R*) and **N**(*R*) are the matrices of Riccati–Bessel and Riccati–Neumann functions.^38^

From the K-matrix, the S-matrix is easily obtained, from which one can extract the individual state-to-state cross sections. We have already published an algorithm that modifies the variable-phase approach to solve that problem, specifically addressing the latter point, and we refer the interested reader to those references for further details.^37,38^

In the present calculations, we have generated a broad range of state-to-state rotationally inelastic cross sections from the interaction of HeHHe^+^ with He. We shall also be comparing the new results with our earlier ones on the HeH^+^ cation with He atoms^40^ to provide a quantitative assessment with a cation already observed in the ISM environment.

Specifically, our present calculations have used up to 26 terms in the multipolar expansion of the initial PES to account for the strong anisotropy of the present system. The radial range of integration has been varied depending on the collision energy considered and has extended from 1.5 to 100 Å, and the number of coupled channels in the coupled equations went up to 22 rotational states. In the global partial wave expansions of the calculations, the range of *J* values from *J* = 0 up to *J*_max_ = 100 was found to be sufficient to reach convergence of the numerical values of the state-to-state opacity functions. The range of energies considered for the cross sections has increased from 0.0001 up to 2260.00 cm^–1^. Transitions have been calculated between *j* = 0 and *j* = 10 with all of the intermediate values taken into account for producing final inelastic cross sections.

Once the state-to-state inelastic integral cross sections are known, the rotationally inelastic rate constants *k*_*j*→*j*′_(*T*) can be evaluated as the convolution of the cross sections over a Boltzmann distribution of the relative collision energies
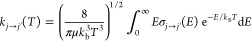
5

where μ is the reduced mass of the system, given by 2.77166 amu, and *k*_B_ is the usual Boltzmann constant. The numerical quadrature was carefully controlled over the required range of computed cross sections, which we shall be reporting in the next section, using numerical polynomial interpolation/extrapolation to reach the necessary cross section threshold values and the values at the highest energies included.

The interplay between the effect of the reduced mass value, appearing in the denominator in the equation above, and the structural strengths of the corresponding PES multipolar coefficients given in Figure 2, has an important effect on the inelastic dynamics since they work in opposite directions in producing the final values of the inelastic rate constants. We shall see from our results that the marked anisotropy of the interaction potential is the property that is mainly driving the large state-changing probabilities we have found for this molecule.

## Results and Discussion

The quantum calculations of the relevant rotationally inelastic cross sections of the present cation were carried out over a broad range of energies expected to be sufficient to describe the lower temperature and lower redshift values at which the existence of the title molecule could occur under early universe conditions.

### Inelastic Cross Sections and Rate Coefficients

**Figure 4:**
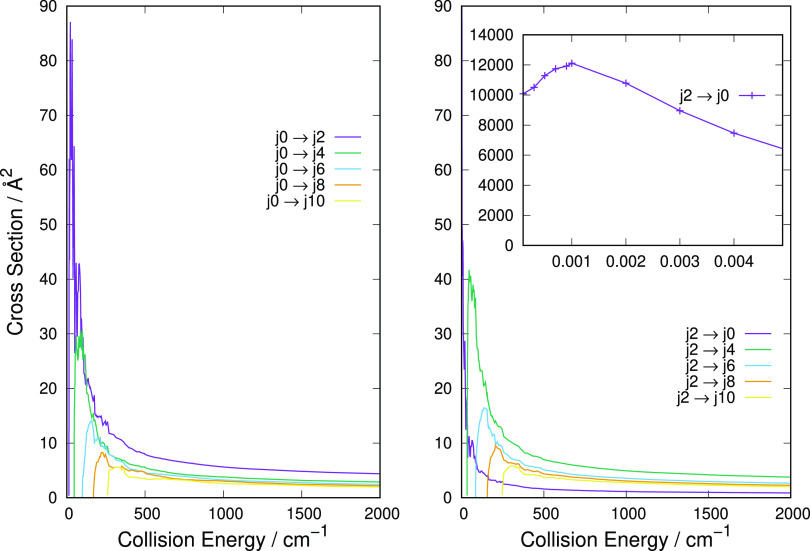
State-to-state computed partial integral cross sections for the inelastic processes that start from the *j* = 0 rotational state (left) and for those processes that originate from the *j* = 2 state (right). The inset in the right panel shows an enlarged view of the Δ*j* = −2 deexcitation cross section between the two lowest levels near the threshold energy.

More specifically, the data shown in Figure 4 report the excitation processes with increasing Δ*j* values, keeping in mind that the symmetry of the present rotor only allows for even values of the rotational quantum numbers, as discussed before. We show there as examples the state-changing processes originating from the *j* = 0 and *j* = 2 states.

When looking at the excitation cross sections from the lowest rotational state of this cation, we notice that, as discussed earlier, the process involving the direct Δ*j* = 2 transition, i.e., the one chiefly activated by the direct dynamical coupling via the multipolar coefficient with λ = 2, is by far the largest inelastic cross section at threshold and remains so up to the highest collision energy we have considered in this study. In that same threshold energy region, we see that the excitation process largely controlled by the direct multipolar coefficient with λ = 4, the one previously seen to be the next more important coupling torque for the present system, is very large and comparable in size to the process discussed before. As the collision energy increases, the latter inelastic cross section with Δ*j* = 4 becomes smaller than the former one (with Δ*j* = 2) but shows a very similar energy dependence. It is also interesting to note in that panel that all of the other inelastic processes, involving increasingly larger changes of the Δ*j* values up to Δ*j* = 8, become increasingly smaller near the threshold region, but at higher energies, they remain comparable in size with the state-changing processes associated with the smallest Δ*j* values. This is an interesting result which suggests that the efficiency of energy uptake via collisions by the present cation could involve a larger range of possible transitions than would be expected from what other ionic systems have shown in our earlier calculations.^40^

The results shown in Figure 4 (right) report a sequence of state-changing processes with transitions from the initial *j* = 2 rotational state of the cation. The specific threshold behavior shown in the inset also indicates that the deexcitation process associated with the Δ*j* = −2 transition is the largest of all processes, remaining more than 2 orders of magnitude larger than all of the others at collision energies within fractions of cm^–1^. The smallest energy gap involved, more significant at the lowest collision energies where the corresponding cross section is by far the largest, is therefore causing larger cross sections. However, as the collision energy moves above the various threshold energies, all of the inelastic cross sections become comparable in size, and the deexcitation process increasingly becomes the smallest cross section in comparison to the excitation transitions from the same *j* = 2 state. This behavior indicates that the increases in the energy spacing between the higher rotor states reduce the interaction time between partners when deexcitation is considered while they increase it for excitation processes. Hence, the adiabatic criterion qualitatively suggests that energy transfer efficiency is best when the forces induced by the collision are on the same time frame as that of the internal motion. Thus, excitation and deexcitation processes change their relative importance depending on the considered range of collision energies in relation to the energy gaps involved (e.g., see ref (41)). This behavior was also found in our earlier work with different cations^42^ and will be discussed when analyzing the inelastic rate coefficients.

**Figure 5:**
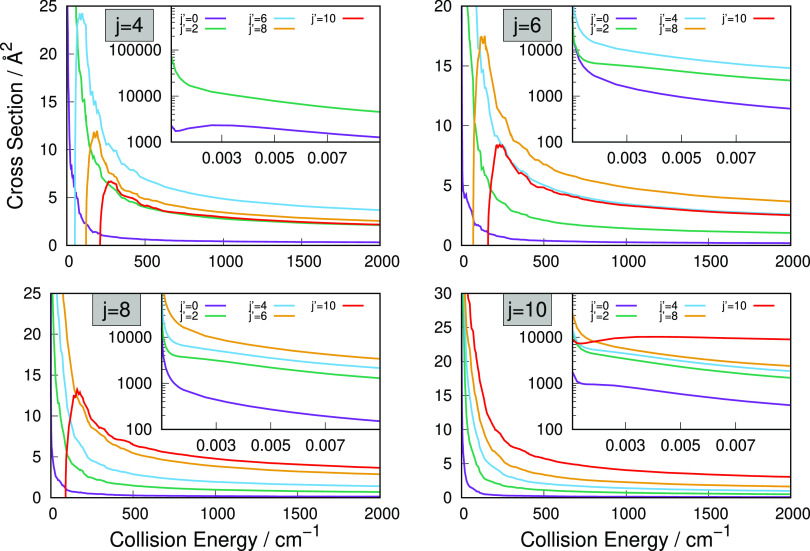
State-to-state computed partial integral cross sections for the excitation and deexcitation processes. The four panels indicate state-changing processes that originate from different initial rotational states of the cation. The top left and top right panels show transitions from the initial *j* = 4 and *j* = 6 states, respectively, while the bottom left and bottom right panels present transitions from the *j* = 8 and *j* = 10 rotational states, respectively. The insets in all panels show the cross section behavior near the threshold energies.

Additional calculations are reported in Figure 5, where we show a more extended range of collision energies for which the state-changing processes have been investigated. The insets in each of the panels indicate the large cross section values found near the corresponding threshold for the deexcitation processes. The initial rotational states considered for each set of calculations increase along the panels, from the top left to the bottom right so that each panel shows both excitation processes and deexcitation processes occurring during collisions from that particular initial state. Such choices help reveal the differences in energy-storing and energy-releasing collisional events when they involve the network of rotational states of this cation. We have seen from the Boltzmann-type populations presented in Figure 3 that the lower rotational states up to *j* = 10 should be significantly populated at the temperature of interest. Although we do not know as yet whether to expect the present molecule to be within local thermal equilibrium (LTE) for rotational states, we shall take it as a qualitative initial indicator for helping us in choosing the most important rotational states to be considered here.

Figure 5 shows that at the thresholds for the opening of the excitation (energy-storing) processes, their cross sections are dramatically larger than those pertaining to the deexcitation (energy-releasing) processes. When the collision energy moves up from a few cm^–1^ to the energies above the inelastic excitation thresholds, and then up to about 2000 cm^–1^, we clearly see that the excitation cross sections remain dominant in size while the deexcitation channels decrease as the energy increases. Such behavior is again in line with the adiabatic criterion mentioned for the previous figure and concerning the interplay between interaction times and involved energy gaps as already discussed in ref (41).

**Figure 6:**
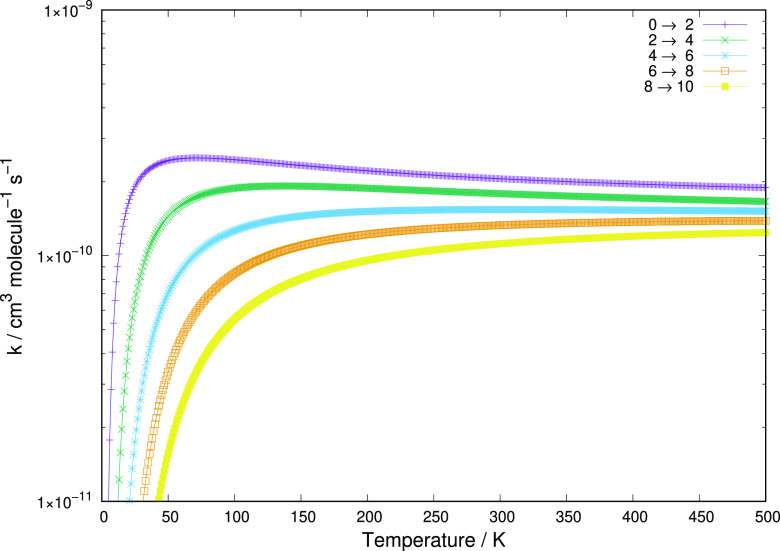
Computed rotationally inelastic excitation rate coefficients associated with the Δ*j* = 2 transitions starting from the *j* = 0 level to the *j* = 8 level.

**Figure 7:**
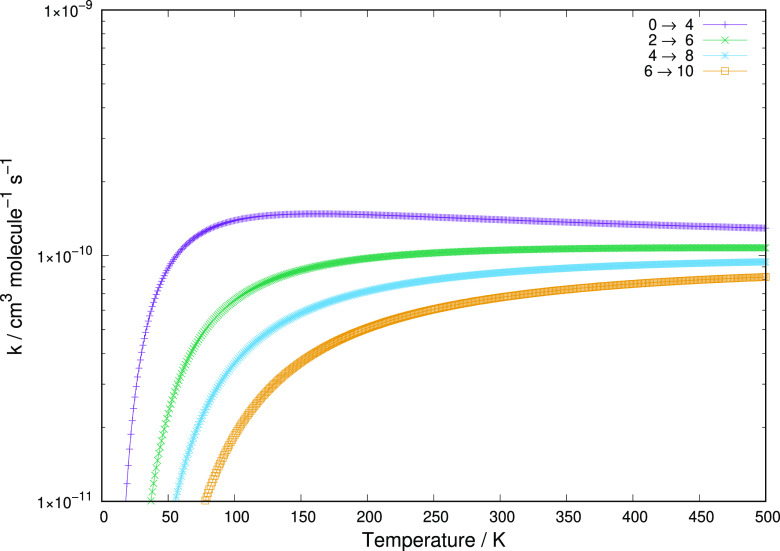
Same computed quantities as in Figure 6, but here showing excitation rate coefficients for the Δ*j* = 4 transitions, starting from the *j* = 0 level to the *j* = 8 level.

**Figure 8:**
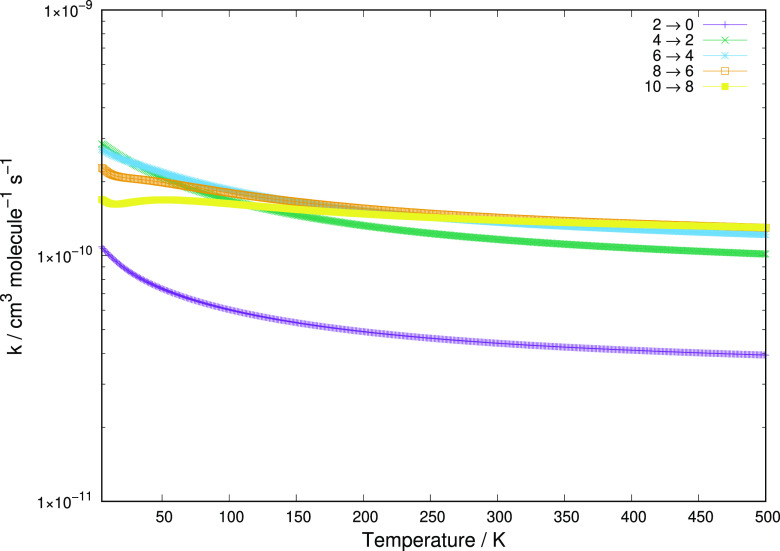
Same computed quantities as in Figure 6, but here showing deexcitation rate coefficients for the Δ*j* = −2 transitions, starting from the *j* = 2 level to the *j* = 10 level.

As mentioned in the previous section, the inelastic cross sections are the starting ingredient for the production of the rate coefficients associated with those state-changing processes over the range of temperatures of interest for kinetic modeling studies. The present calculations were carried out for the rotational states discussed in the previous section, and the resulting rate constants are given in Figures 6–8. Because of the specific energy-dependence behavior we found for the inelastic cross sections, the actual numerical quadrature to obtain the corresponding rate coefficients from the lowest temperatures and up to 500 K was very carefully controlled to make sure that all values, at all temperatures, were numerically converged within a few percents and that the final rate values were stabilized.

The data reported in Figure 6 clearly show that the relative size of the computed rate coefficients decreases with the increase of the energy gaps between different transitions. The largest transition energy, associated with the *j* = 8 to *j* = 10 rotational excitation, is nearly 1 order of magnitude larger than the one for the *j* = 0 to *j* = 2 rotational excitation. Accordingly, the latter rate constant is on average about twice as large as the former. This inverse relation between size and energy gap uniformly applies to all of the rate coefficients shown in the figure. Furthermore, the marked anisotropy discussed earlier for the employed PES is also causing here that the range of values of all rate coefficients remains fairly large at the temperatures considered, once beyond their relative threshold values, so that the size differences just mentioned are fairly small in relative terms.

We have mentioned earlier that the direct coupling potential for the Δ*j* = 4 transitions is close in strength and radial range to the one that applies the direct rotational torque for the Δ*j* = 2 transitions. As a result of this feature, we therefore see that the excitation rate constants reported in Figure 7 and showing the excitation processes for the Δ*j* = 4 state-changing collisions are not significantly smaller than those for the Δ*j* = 2 excitations. At the highest temperatures shown in Figures 6 and 7, we see that the values in Figure 7 are only a few percents smaller than those presented in Figure 6. We can therefore argue that collisional excitation processes for the title cation in collision with He atoms would efficiently cover a fairly broad range of internal state excitations, thereby contributing to the internal energy-storing step under expected conditions at the time of the recombination era.

Another set of useful comparisons can be seen from the sampling of deexcitation rate coefficients reported in Figure 8.

The deexcitation rate coefficients reported in Figure 8 clearly show that the rate values increase as the initial rotational state of the cation increases, with the deexcitation process from the lowest initial state possible (*j* = 2) being that with the smallest rate coefficient. These findings are in the opposite order with respect to the excitation processes discussed in Figures 6 and 7, where the excitation rates from the lower levels were the larger ones. This feature has to do with the opposite role of the energy gap value, in either excitation transitions or deexcitation transitions, with respect to the dynamics being more or less impulsive after collision depending on the value of the residual/released relative kinetic energy. Thus, the deexcitation processes remain efficient and are larger when more internal energy is being released into the relative kinetic energies of the partners (see again ref (41) for an earlier discussion of this point).

### Further Indicators from Inelastic Rate Coefficients

**Figure 9:**
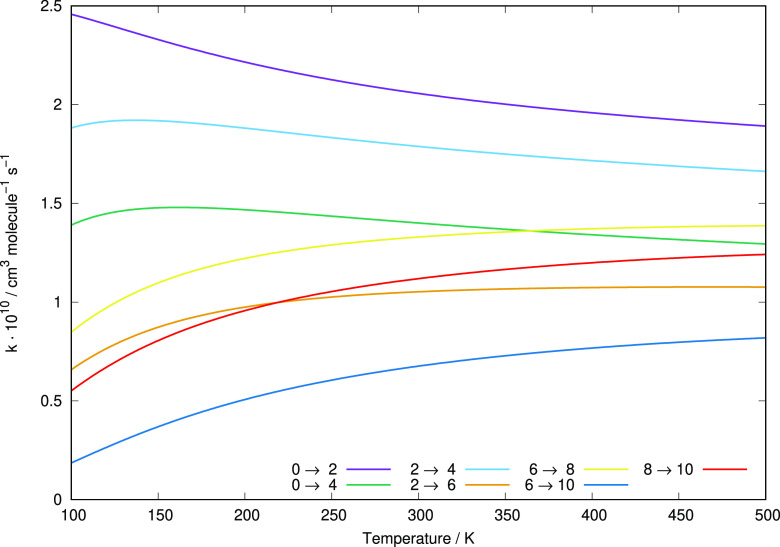
Enlarged view of the computed excitation rate constants from different initial levels covering the range examined in this work and involving both Δ*j* = 2 and Δ*j* = 4 transitions.

To provide another comparative view on the behavior of the collisional rotational state-changing dynamics presented in previous figures, we report in Figure 9 an enlarged comparison of a subset of excitation rate constants from different initial rotational states of the cation, undergoing both Δ*j* = 2 and Δ*j* = 4 excitations. We clearly see there that, in spite of the large differences in the energy gaps between the levels (e.g., see the data in Figure 3), the efficiency of the collisional excitations does not change much over the range of temperatures examined. The range in size differences between the lowest excitation rate coefficient (that for the *j* = 0 to *j* = 2 process) to the one involving Δ*j* = 4 transitions between the most highly excited levels considered in this study (the *j* = 6 to *j* = 10 case) is on average a factor of 2 at most. It is therefore the strong anisotropic potential coupling discussed earlier, and the similar strength of the two anisotropic terms for λ = 2 and λ = 4 that dominates the outcomes of the inelastic dynamics, hence limiting the effects from the energy gaps in the excitation processes.

**Figure 10:**
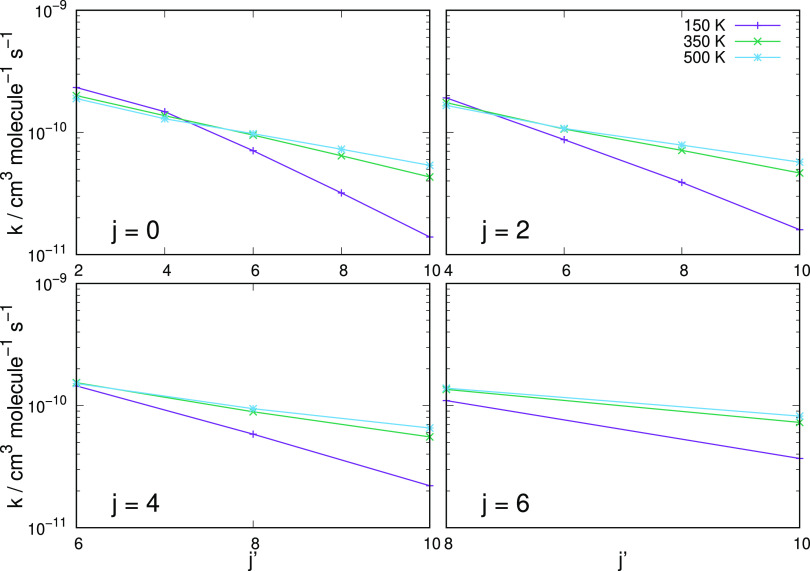
Behavior of the computed excitation rate constants at three different temperatures and for transitions that start from different initial rotational states. Each panel reports all three different temperatures for a given initial state of the cation.

The data shown in Figure 10 present a different way of assessing the rotational excitation efficiency of the present system. We show results for three different temperature values, while each panel in turn reporting excitation rate coefficients for multiple transitions originating from a different level in each panel. The following considerations can be made:

(i) although we are considering transitions from Δ*j* = 2 to Δ*j* = 10, we see that the rate constant values change rather little along the four panels, especially when the higher temperatures are considered;
(ii) only at the lowest temperature a significant drop in rate constant value occurs as Δ*j* increases: we have seen from the previous figures reporting the individual rates that the *T* dependence at the lower temperatures varies markedly as Δ*j* changes, hence the present effect;
(iii) the slow dependence of the inelastic rate coefficients on temperatures at higher *T* values suggest that the efficiency of the energy transfer processes remains large over a broad range of environmental situations, hence underlying the good energy-storing efficiency expected from the present molecule under early universe conditions.

**Figure 11:**
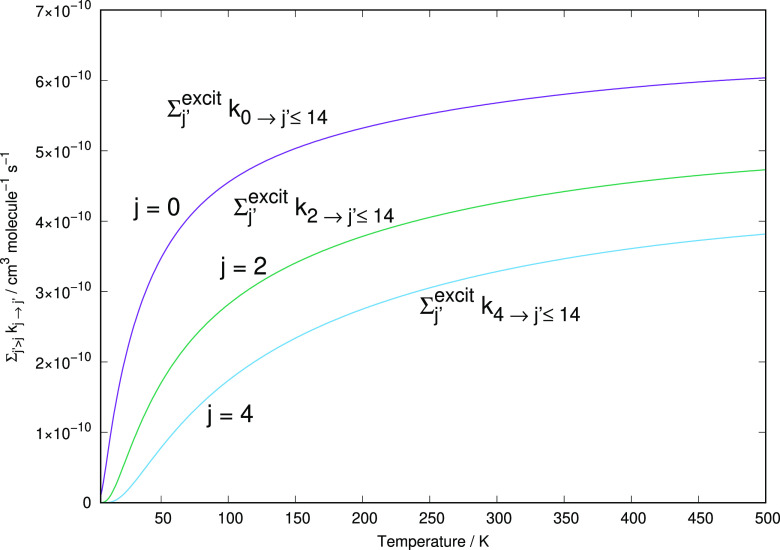
Computed excitation rate coefficients summed over all accessible final levels and starting from the lowest three rotational states of the present cation. Notice the enlarged scale of the ordinates.

An additional set of indicators is shown by the data in Figure 11. The excitation rate coefficients are now summed over all of the accessible final states as the temperature values span the range discussed in this work. This quantity therefore provides a sort of global indicator of the excitation efficiency shown by the HeHHe^+^ in collision with neutral He. Three different situations are shown in the figure, with different initial rotational states of the target being selected: *j* = 0, *j* = 2, and *j* = 4, with the inclusion of all available excitation transitions from each of these levels.

All of the summed rate coefficients turn out to be fairly close in value, with the processes initiated with the cation in its ground-state rotational level being the largest, as should be expected from the dominance of the *j* = 0 to *j* = 2 transition seen in the previous sections. We further notice that the changes in size from the smallest to the largest quantity presented in Figure 11 are rather small, once more suggesting the overall large efficiency of the present excitation processes at the temperatures of interest.

As we have mentioned in the Introduction section, the possible presence of HeHHe^+^ is also linked to the presence of the recently observed HeH^+^ polar cation. Some of the paths to its formation and to the further formation of other (He,H)-containing molecules have been recently discussed in ref (44). We have also outlined some further possibilities in the Introduction section and will discuss them more specifically in future work. Since neutral He and neutral H atoms can interact with either of the above ionic molecules, we thought it to be of interest to compare the efficiency of the energy transfer processes involving rotational states of both of them when colliding with surrounding hydrogen atoms, especially since data already exist for the case of HeH^+^ in collision with H from the earlier work reported in refs (43) and (40).

**Figure 12:**
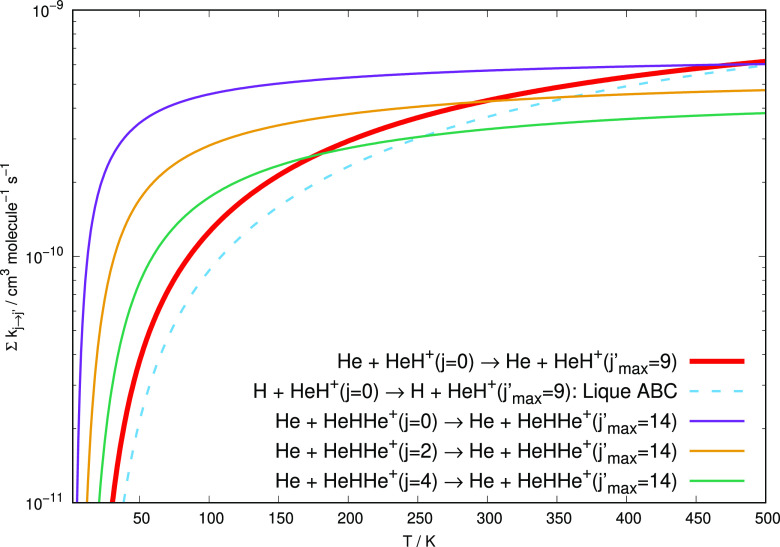
Comparison of the behavior of the summed excitation rates for the HeHHe^+^ and the HeH^+^ systems in collision with He and H atoms. The dashed thin line reports the calculations from ref (43) for collisions of H atoms with HeH^+^, while the thick solid line reports our earlier calculations from ref (40) on the HeH^+^ collisions with He atoms. The present results for the HeHH^+^ in collision with He atoms are given by the three thin solid lines of different colors.

We present a comparison in Figure 12, where different cases for the computed global, inelastic rate coefficients for both HeH^+^ and HeHHe^+^ in collision with He atoms are shown. Earlier results for the case of HeH^+^ colliding with H atoms are also reported.^43^

The following comments can be made from a perusal of the data:

(i) In the lower range of temperatures, up to about 250–300 K, the efficiency of the rotational energy transfer dynamics for the case of the present molecular ion HeHHe^+^, when in one of its lowest rotational states *j* = 0 or *j* = 2, is much larger than that found for HeH^+^. These states are also the most probable initial states at the expected temperature conditions in the ISM;
(ii) The collisional inelastic rate constants involving either H or He atoms interacting with HeH^+^ are becoming of the same size as those for HeHHe^+^ only at the highest temperatures considered in the present study, while remaining smaller at the lower temperatures.

The above results indicate that the present cation, if in existence where HeH^+^ has been already found to be present,^12^ would be a more efficient participant to the energy-storing mechanisms induced by collisions with abundant species, like He and H, present in the same environment. This is an interesting conclusion, suggesting that including such species within enlarged evolutionary models of the chemistry in the early universe would be a useful improvement (e.g., see ref (13)).

From the variety of estimated baryon densities in the early universe discussed earlier, in fact, we already know that the baryon density *n*_b_ is proportional to the redshift value *z* via the relationship: (1 + *z*)^3^ (see ref (21)). Hence, we can say that for values of *z* varying between 200 and 5000, the corresponding *n*_b_ values would be varying between about 10^–1^ cm^–3^ and about 10^3^ cm^–3^. Such values would therefore bracket the likely range of densities for the He partner as one of the most abundant species that can be expected to exist at the recombination era of the early universe conditions, as discussed in ref (21). To better understand the variations that such density values would have on the collisional astronomical times of the present species with the He atoms, it is convenient to introduce another useful, global indicator by defining a quantity, called τ, obtained from the summed collisional relaxation rate coefficients multiplied by the available density estimates of the environment under consideration
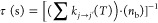
6

This quantity, given in units of s, can be obtained using the two extreme values of *n*_b_ mentioned earlier and summing over all our calculated rate coefficients *k*_*j*→*j*′_(*T*) discussed in the previous section. We therefore find that the purely collisional relaxation times provided by τ, depending on the estimated baryon density range in the early universe models, can vary rather dramatically over more than 4 orders of magnitude from 10^10^ s at the lowest redshift values (*z* = 200) to about 10^6^ s at the higher redshift values of 5000. This result further underscores in more quantitative terms the role of collision-driven energy exchanges in relation to the actual density conditions in which the molecular partners are operating.

## Conclusions

One of the aims of the present study has been to consider the energy dissipation role, through rotational energy transfer paths, of a possible new entry among the early universe molecules, the HeHHe^+^ cation, interacting with He atoms under the expected conditions suggested by the existing modeling studies (e.g., see ref (21)). Although this cation has not yet been observed, its likely formation routes under the astronomical conditions have been studied in the recent literature (e.g., see refs (23, 24)) where various possibilities for its formation and energy dissipation paths were surmised.

We have pursued this specific aspect through direct quantum dynamics calculations and have obtained the potential energy surface for the interaction between this cation, treated in a first instance as being at its equilibrium bond distances (a rigid rotor target), and the He neutral atom using high-quality ab initio treatment. We have then employed it to generate the quantum inelastic cross sections for rotational excitation processes over a broad range of rotational states and further evaluated the corresponding inelastic rate coefficients up to about 500 K for the interstellar conditions. The results obtained have been illustrated in detail in the previous sections and indeed suggest that the strong anisotropy of the interaction potential is responsible for inducing rather large inelastic, rotational state-changing cross sections right from near-threshold collision energies, thereby generating the corresponding inelastic rate constants as being unexpectedly large for this cation and under the examined temperature conditions.

We have also shown various dynamical indicators and further compared the present findings with earlier results involving both He and H as collision partners of a polar cation present after the recombination era: the HeH^+^. This comparison indicates that HeHHe^+^ yields larger inelastic rate coefficients under similar conditions as those where the HeH^+^ was studied, thus surmising that the inclusion of this molecule into the chemical networks modeling the kinetic evolution of the early universe would provide a more realistic description of the actual energy dissipation processes, which could occur under those conditions.

Although the present molecular cation has no permanent dipole moment at its equilibrium geometry, the existence of an asymmetric stretching mode with a substantial transition dipole as discussed in refs (23, 24) could provide another collisional route to energy transfer dynamics. This possible option will be further discussed in future work in preparation in our laboratory.

## Supporting Information Available

Multipolar coefficients for the Legendre expansion of the new rigid rotor PES for the HeHHe^+^ interaction with He are provided via a Fortran program routine and data files providing the computed inelastic rate coefficients for the rotational excitation of the title molecule in collision with He atoms (ZIP)
