# Supplementary material for: Dynamics of HeHHe+ Rotational State Changes Induced by Collision with He: A Possible New Path in Early Universe Chemistry
Source: J Phys Chem A. 2021 Apr 26;125(17):3748–59. doi: 10.1021/acs.jpca.1c01820 (PMC8154607; doi:10.1021/acs.jpca.1c01820)
Supplement: Supplementary file 1 — jp1c01820_si_001.zip [file jp1c01820_si_001.zip › Supporting Information/ S1-multipolar radial coefficients/Title and Authors.pdf]

Dynamics of $HeHHe^+$ Rotational State Changes Induced by Collision with He: A Possible New Path in Early Universe Chemistry

L. González-Sánchez,[†] E. Yurtsever,[‡] R. Wester,[¶] and F. A. Gianturco[*,¶]

†Departamento de Química Física, University of Salamanca

Plaza de los Caídos sn, 37008, Salamanca, Spain

‡Dept. of Chemistry, Koc University, Rumelifeneri Yolu, Sariyer, TR, 34450 Istanbul,Turkey

¶Institut fur Ionen Physik und Angewandte Physik, Leopold-Franzens-Universitat, Technikerstrasse 25, 6020, Innsbruck, Austria
