# Supplementary material for: Dynamics of HeHHe+ Rotational State Changes Induced by Collision with He: A Possible New Path in Early Universe Chemistry
Source: J Phys Chem A. 2021 Apr 26;125(17):3748–59. doi: 10.1021/acs.jpca.1c01820 (PMC8154607; doi:10.1021/acs.jpca.1c01820)
Supplement: Supplementary file 1 — jp1c01820_si_001.zip [file jp1c01820_si_001.zip › Supporting Information/Title and Authors .pdf]

Dynamics of $HeHHe^+$ Rotational State Changes Induced by Collision with He: A Possible New Path in Early Universe Chemistry

L. González-Sánchez,[†] E. Yurtsever,[‡] R. Wester,[¶] and F. A. Gianturco*,[¶]

†*Departamento de Química Física, University of Salamanca*
Plaza de los Caídos sn, 37008, Salamanca, Spain

‡*Dept. of Chemistry, Koc University, Rumelifeneri Yolu, Sariyer, TR, 34450 Istanbul, Turkey*

¶*Institut fur Ionen Physik und Angewandte Physik, Leopold-Franzens-Universitat, Technikerstrasse 25, 6020, Innsbruck, Austria*

* corresponding author : francesco.gianturco@uibk.ac.at
